# Similarity of morphological composition and developmental patterning in paired fins of the elephant shark

**DOI:** 10.1038/s41598-017-10538-0

**Published:** 2017-08-30

**Authors:** Cyrena Riley, Richard Cloutier, Eileen D. Grogan

**Affiliations:** 1 Laboratoire de Paléontologie et Biologie évolutive, Université du Québec à Rimouski, Rimouski, Québec G5L 3A1 Canada; 2 Biology Department, Saint Joseph’s University, Philadelphia, Pennsylvania 19131 USA

## Abstract

Jawed vertebrates, or gnathostomes, have two sets of paired appendages, pectoral and pelvic fins in fishes and fore- and hindlimbs in tetrapods. As for paired limbs, paired fins are purported serial homologues, and the advent of pelvic fins has been hypothesized to have resulted from a duplication of the developmental mechanisms present in the pectoral fins, but re-iterated at a posterior location. Developmental similarity of gene expression between pectoral and pelvic fins has been documented in chondrichthyans, but a detailed morphological description of the progression of paired fin development for this group is still lacking. We studied paired fin development in an ontogenetic series of a phylogenetically basal chondrichthyan, the elephant shark *Callorhinchus milii*. A strong similarity in the morphology and progression of chondrification between the pectoral and pelvic fins was found, which could be interpretated as further evidence of serial homology in paired fins, that could have arisen by duplication. Furthermore, this high degree of morphological and developmental similarity suggests the presence of morphological and developmental modules within paired fins, as observed in paired limbs. This is the first time morphological and developmental modules are described for the paired fins of chimaeras.

## Introduction

Amongst the evolutionary novelties associated to the origin of jawed vertebrates (including “placoderms^**†**^,” “acanthodians^**†**^,” chondrichthyans, and osteichthyans) figure the simultaneous presence of paired appendages (pectoral and pelvic fins or limbs)^1^. The morphology and development of vertebrate paired appendages have been intensively investigated^2, 3^ with great interest focused on the rise of the tetrapod condition^4–6^. Tetrapod limbs arose through modifications of the paired fins of sarcopterygian fish (derived osteichthyans) during the Upper Devonian, some 380 Ma.^2, 4, 5^. Tetrapod limbs are assumed to be serially homologous structures resulting from the duplication of the underlying developmental program of a modular structure at a novel location and time^2, 7, 8^. Paired limbs are also considered morphological and developmental modules^8, 9^ because of their anatomical and developmental similarities. Modules, such as the paired limbs^8, 9^, are units that comprise numerous interacting parts that are functionally integrated, hierarchically organized and capable of independent evolutionary trajectory^9, 10^.

Comparatively, the morphology and development of paired fins are not as well characterized, especially in groups occupying a basal phylogenetic position. For example, in chondrichthyans, despite the molecular evidence supporting the shared phenotype and development of paired appendages^11–13^, fundamental morphological data are still lacking, notably concerning the formation of paired fins. Morphogenesis can provide valuable information on the origin and evolution of paired fins^14^, and developmental series are useful to better understand the formation of morphological structures^15, 16^. The morphology of paired fins in chondrichthyans (Elasmobranchii and Holocephali), which are living gnathostomes occupying a basal phylogenetic position^17^, is generally accepted as reflecting a comparatively less derived condition compared to osteichthyans (including Actinopterygii and Sarcopterygii)^14, 18^, since pectoral and pelvic fins in some osteichthyans have different morphologies and developmental patterns^5, 19^. However, modern elasmobranchs (sharks, skates and rays) also exhibit a diverging morphology between pectoral and pelvic fins^5^, and thus have often been overlooked in morphological studies of paired fins. Within extant chondrichthyans, chimaeras (Holocephali) occupy a basal phylogenetic position^20^, possess the slowest evolving genome of vertebrates currently sequenced^21^, and retain a greater number of less derived genes compared to osteichthyans^22^. Although specific structures (cranium, branchial arches) appear to exhibit a derived condition in the Holocephali^16, 23, 24^, this group may provide perspective on chondrichthyan ancestral conditions^23^. Thus, the Holocephali represents ideal extant chondrichthyans in which to investigate development, as there is a need for more embryological information concerning this group^25^. Here we use a growth series of the elephant shark *Callorhinchus milii* as representative of the oldest and most basal chimaera family, the Callorhinchidae^17, 26^. The main goals of this study are 1) to provide a description of the morphology and chondrification pattern of the pectoral and pelvic fins of *C*. *milii* and 2) to verify whether pectoral and pelvic fins have similar morphology and similar chondrification patterns.

## Results

The pectoral fin of *C*. *milii* consists of two proximal basal elements (propterygium and metapterygium) whereas the pelvic fin consists of a single basal element (basipterygium, including the metapterygium). Both pectoral and pelvic basal elements articulate distally with rows of proximal, middle and distal radials. Only the progress of chondrification is described, since no mineralization of endoskeletal elements was observed. Radials are numbered antero-posteriorly starting with number one.

### Developmental progression of the pectoral fins

**Figure 1:**
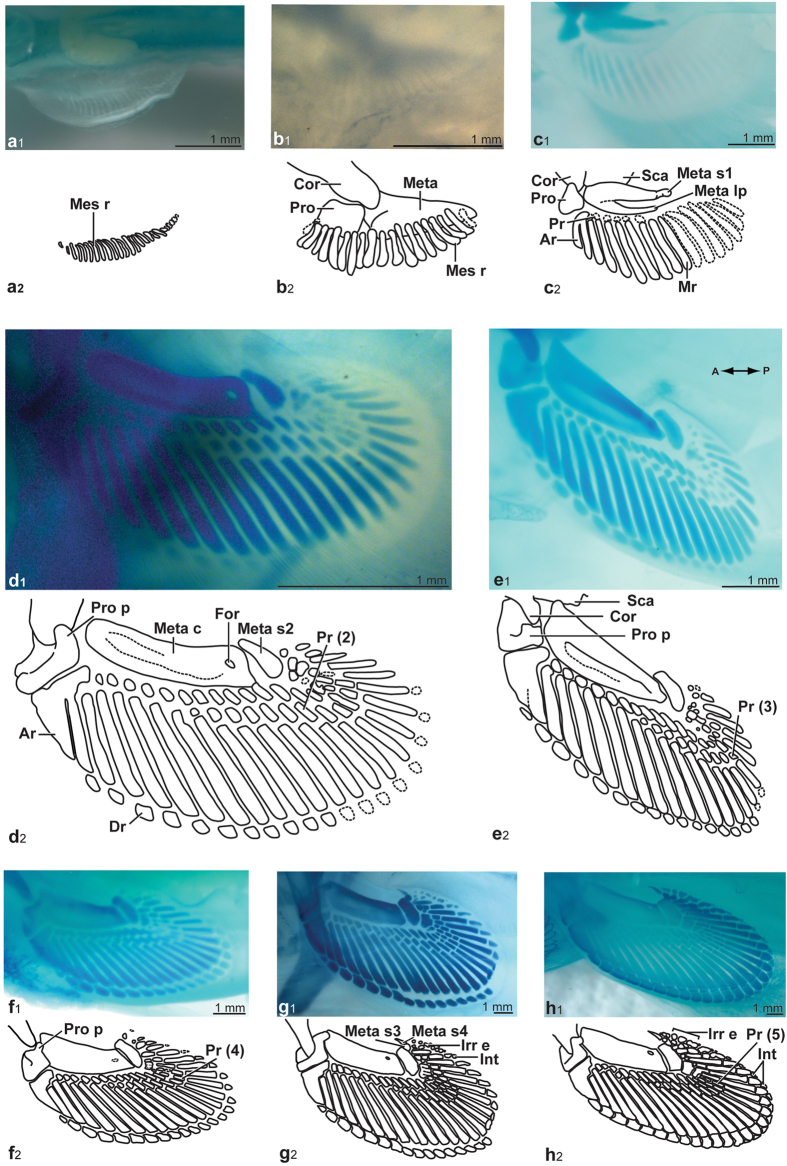
Progression of chondrification in *Callorhinchus milii* pectoral fins, photographs and drawings of endoskeletal structures. (**a**) ANSP 174694, stage 29. (**b**) ANSP 174667, stage 30. (**c**) ANSP 174661, stage 31. (**d**) ANSP 174688, stage 32. (**e**) ANSP 174691, stage 33. (**f**) ANSP 174692, stage 34. (**g**) ANSP 174663, stage 35. (**h**) ANSP 174675, stage 36. Abbreviations: **Ar**, anterior radial element; **Cor**, coracoid; **Dr**, distal radial; **For**, foramen; **Int**, interdistal; **Irr e**, irregular element; **Mes r**, mesenchymatous rod; **Meta**, metapterygium; **Meta c**, metapterygial complex; **Meta lp**, metapterygial lateral process; **Meta s1**, first metapterygial segment; **Meta s2**, second metapterygial segment; **Meta s3**, third metapterygial segment; **Meta s4**, fourth metapterygial segment; **Mr**, middle radial; **Pr**, proximal radial; **Pr** **(** **2** **)**, second row of proximal radial; **Pr** **(** **3** **)**, third row of proximal radial; **Pr** **(** **4)**, fourth row of proximal radial; **Pr** **(** **5)**, fifth row of proximal radial; **Pro**, propterygium; **Pro p**, propterygium process; **Sca**, scapula. All specimens are cleared and stained; cartilages are stained in blue.

Stage-29 pectoral fins are becoming distinct from the lateral finfold and have a rounded shape (Fig. 1a). A maximum of 25 mesenchymatous rods span the entire length of the fins, and their distal extremities are rounded. These rods are lined next to one another in a slight inward curve. All rods have similar width, and rods placed at the anterior and posterior limits of the fins are smaller than those located in the middle.

In stage-30 pectoral fins (Fig. 1b), the propterygium appears as a squarish structure positioned proximally to the metapterygium, near the coracoid. The metapterygium is an elongated triangular structure that appears also closely associated with the coracoid. The ceratotrichia are present along the distal-most fin margin of the pectoral fins.

In stage-31 specimens (Fig. 1c), the coracoid articular process is located between the propterygium anteriorly and the metapterygium posteriorly. The propterygium is approximately triangular in shape, and its postero-lateral edge is located near the proximal extremities of middle radials 1–2 which are fused proximally to one another. A condensation of cells is found in continuity with the forming metapterygium, appearing as an elongated mass lateral to the medial margin of the developing metapterygium (Fig. 1c). This condensation could be interpreted either as a lateral process of the metapterygium, or as a distinct basal, the mesopterygium. Herein, it is interpreted as the lateral process of the metapterygium, as there is evidence that it continues to condense and unite with the rest of the metapterygium in a proximo-distal direction. The longest stage-31 specimen (ANSP 174661; Fig. 1c) reveals a rounded element distal to the long axis of the metapterygium proper with a paler cartilaginous bridge between them; this establishes the first of the smaller cartilaginous elements contributing to the metapterygial axis. The first rows of proximal and middle radials are beginning to chondrify following an antero-posterior sequence from the mesenchymatous rods, where mesenchymatous rods 1–2 gives rise to median radials 1–2, and mesenchymatous rod 3 gives rise to proximal and middle radials 3 (Fig. 1c). Proximal radials are less advanced in their chondrification compared to middle radials.

The pectoral fins of stage-32 specimens display a propterygium with a rounded process marking the lateral edge; the distal edge is slightly concave and articulates with the developping anterior radial element (Fig. 1d). The propterygium is closer to and articulates with the coracoid. The progressive condensation and differentiation of the metapterygium and its lateral process is continuing further distally and their distal regions have fused together. In addition, the fins of the largest specimen (ANSP 174688) have a propterygium process with a thick ridge on its lateral edge (Fig. 1d). The first metapterygial segment has completely fused with the distal edge of the metapterygium, with the foramen remaining between them marking the position of their original separation (Fig. 1d). Therefore, the metapterygial complex (metapterygium, lateral process, first metapterygial segment) now forms a single structure, with a foramen remaining distally. A new element is present distally, which is interpreted as a second metapterygial segment based on its position within the metapterygial axis. This segment is crescent-shaped and longer than wide. The concave proximal edge of the second metapterygial segment articulates with the distal edge of the metapterygial complex. All radials present are more darkly stained in the anterior part of the fin than in the posterior part (Fig. 1d). A second row of proximal radials is now present between the first row of proximal radials and the middle radials, which may have chondrified as single structures or may result from a division (remodeling) of the proximal extremity of the middle radials. The second row proximal radials, present in the more posterior part of the fin, are chondrifying following an antero-posterior direction. The formation of the anterior radial element is progressing, with the first two middle radials (radials 1 and 2) fused together with purported proximal radials 3–4; they also appear to be fused with the proximal region of the third median radial. Distal radials 1–2 do not appear to be present, whereas distal radials 3–13 are chondrified, but very lightly stained. All radials (proximal, middle, distal) chondrify following an antero-posterior direction of formation (Fig. 1d).

At stage 33, the propterygium has transformed into a thick squarish-structure with protruding squared processes (Fig. 1e). The second metapterygial segment has lengthened and shows clearly defined edges, and is located closer to the distal edge of the metapterygium. The middle radials have become well-defined rectangular structures, more darkly stained. Proximal radials located in the anterior part of the fin are more darkly stained. Distal radials are also chondrifying following an antero-posterior direction. In the largest specimen (ANSP 174691), a third row of proximal radials has formed (Fig. 1e). There is intraspecific variation in the number of elements forming the anterior radial element. In specimen ANSP 174682, the right fin has median radials 1–3 fused together with proximal radials 3–4, whereas the left fin has only middle radials 1–2 that are fused with proximal radials 3–4. Also, there appears to be a small distal radial 1 present in the right fin, but not in the left. Development is not as advanced in the smallest stage-33 specimen (ANSP 174659), and the second metapterygial segment can be seen chondrifying distally from the metapterygial complex.

The pectoral fins of the smallest and largest stage-34 specimens (ANSP 174711, 174712) are not properly stained. In stage-34 specimen ANSP 174692, the protruding squared-processes of the propterygium are thicker and articulate postero-medially with the coracoid (Fig. 1f). The medial edge of the propterygium and the proximal edge of the metapterygial complex now tightly articulate together. The distal edge of the propterygium is slightly concave in order to articulate with the convex edge of the anterior radial element. The metapterygial complex and the second metapterygial segment are wider throughout their length and articulate together. The distal edge of the second metapterygial segment is concave and articulates with the proximal extremity of the fused first row of proximal radials in the posterior section of the fin. Middle radials and the first, second and third rows of proximal radials are more darkly stained. A fourth row of proximal radials is present in the posterior section of the fin. Distal radials are chondrifying following an antero-posterior direction (Fig. 1f).

The development of pectoral fins is almost completed in stage-35 specimens (Fig. 1g). A total of 27 middle radials is present. All distal radials have chondrified and small irregular-shaped elements, with no apparent regular segmentation, have appeared at the medio-distal edge of the fin. The coracoid now principally articulates with the propterygium. The distal edge of the second metapterygial segment articulates with a small rounded element, interpreted as a third metapterygial segment. This third metapterygial segment articulates distally with another element, triangular-shaped and pointing medially, which is interpreted as a fourth metapterygial segment. There can be up to five metapterygial segments present posterior to the metapterygial complex, documenting intraspecific variation among specimens of the same stage.

Several first row proximal radials, articulating with the second metapterygial segment, have fused together; their identity vary from first row proximal radial 18 to 23. In larger specimens, the interdistals are now present up to distal radials 12–13, and have become more triangular in shape. The irregular elements are also more numerous. In the smallest stage-35 specimen (ANSP 174663), small cartilaginous elements, interpreted as interdistals, are present between distal radials 24–26 (Fig. 1g).

The pectoral fins of stage-36 specimen (ANSP 174675) form a tightly articulated and compact structure (Fig. 1h). More proximal radials have fused together including four proximal radials of the second row (18–22) articulating with fused first row proximal radials 18–23. Second and third row proximal radials may appear longer because of fusion between the second, third and fourth row radials. There is a fifth row of proximal radials present within the posterior region of the fin. An interdistal appears to be separating from distal radial 14. Irregular elements are larger and fill the space between middle radial 27 and the last metapterygial segment, which is longer, thinner and points medially (Fig. 1h).

### Developmental progression of the pelvic fins

**Figure 2:**
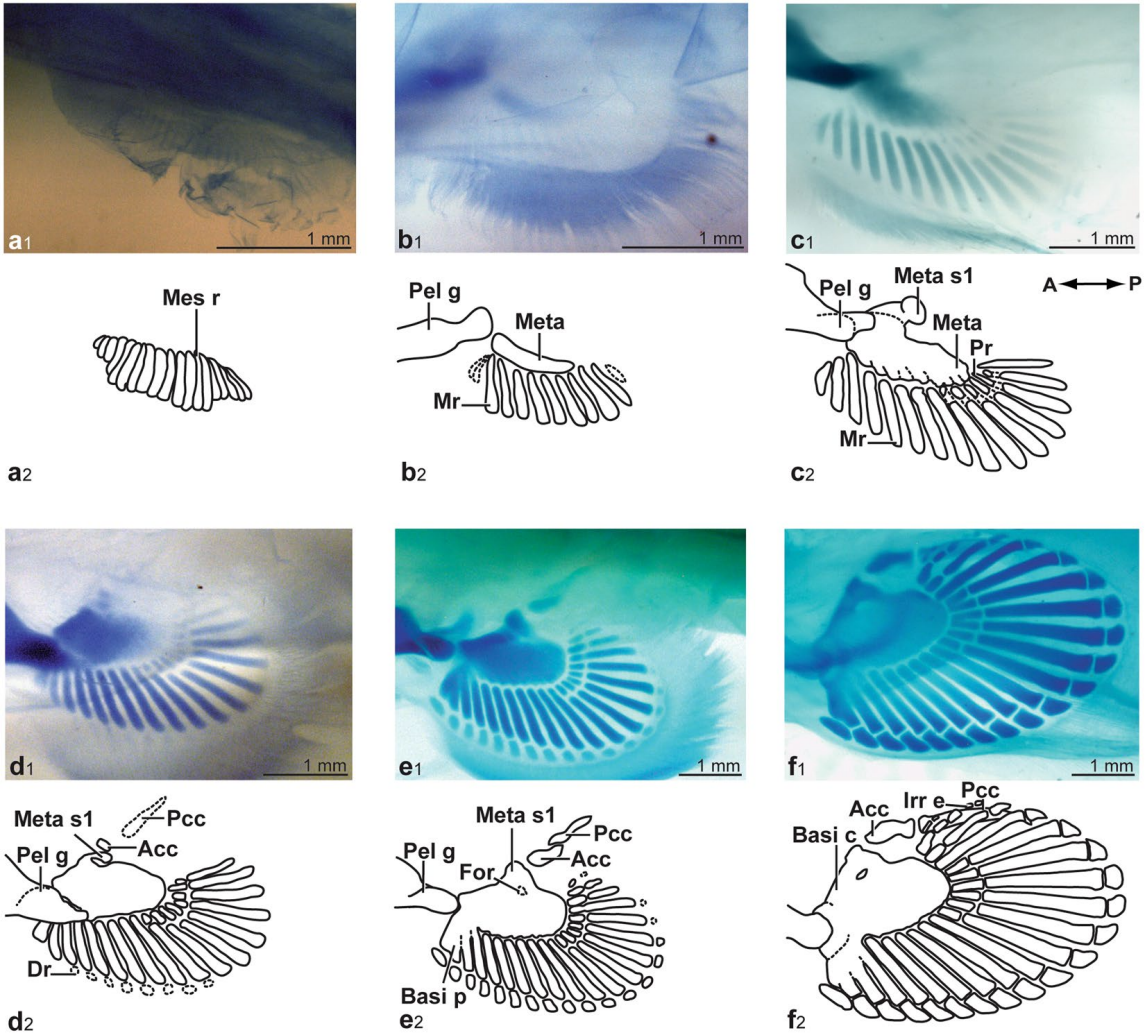
Progression of chondrification in *Callorhinchus* *milii* pelvic fins, photographs and drawings of endoskeletal structures. (**a**) ANSP 174690, stage 29. (**b**) ANSP 174661, stage 31. (**c**) ANSP 174688, stage 32. (**d**) ANSP 174682, stage 33. (**e**) ANSP 174692, stage 34. (**f**) ANSP 174675, stage 36. Abbreviations: **Acc**, anterior clasper cartilage; **Basi c**, basipterygial complex; **Basi p**, basipterygial process; **Dr**, distal radials; **For**, foramen; **Irr e**, irregular element; **Mes r**, mesenchymatous rod; **Meta**, metapterygium; **Meta s1**, first metapterygial segment; **Mr**, middle radial; **Pcc**, posterior clasper cartilage; **Pel g**, pelvic girdle; **Pr**, proximal radial. All specimens are cleared and stained; cartilages are stained in blue.

The general external appearance of the stage-29 pelvic fins is similar to that of the pectoral fins at the same stage. A maximum of 19 mesenchymatous rods are present, with those located at the anterior and posterior limits of the fins nearly half the length of those in the middle (Fig. 2a). No new information can be gathered for the pelvic fins of stage-30 specimens. In stage-31 specimen ANSP 174661, a large cartilaginous mass is forming, near the medial edge of the pelvic girdle (Fig. 2b). This mass is interpretated as a single basal, the metapterygium. The metapterygium is darkly-stained anteriorly and articulates laterally with the mesenchymatous rods. Similarly to the condition observed within the pectoral fins, pelvic basal elements chondrify before radials.

In the pelvic fins of the longest stage-32 specimen (ANSP 174688), the metapterygium is positioned more closely to the pelvic girdle (Fig. 2c). The metapterygium has elongated proximo-distally. A rounded cartilaginous knob is present medially to the metapterygium, and is interpreted as the first metapterygial segment. The lateral edge of the metapterygium has ridges articulating with the adjoining middle and proximal radials. Middle radials 1–3 appear to start to fuse at their proximal extremities and with the anterior edge of the metapterygial complex, beginning the formation of the basipterygial process. Middle radials 2–7 are more darkly stained than those located posteriorly and than middle radial 1. Lightly-stained proximal radials, located in the middle part of the fin, are chondrifying as suggested by the alcian staining. The original mesenchymatous rods from which the radials chondrify can still be observed associated with middle radials 8–14, and proximal and middle radials are still united by mesenchymatous bridges (Fig. 2c).

In stage-33 specimens, (ANSP 174682, 174691), the metapterygium loosely articulates with the pelvic girdle (Fig. 2d). The distal edge of the metapterygium is fusing with the first metapterygial segment, which still has a knob-like appearance. Nearly all middle radials are differentiated, with those at the posterior edge of the fin more lightly stained. Proximal radials are chondrifying following an antero-posterior direction. Anterior distal radials are chondrifying as lightly-stained elements with a diffuse outline. In the pelvic fins of ANSP 174691, a rounded structure is forming distally to the first metapterygial segment, representing the second metapterygial segment. This second segment forms the anterior clasper cartilage (Fig. 2d) whereas the posterior clasper cartilage is forming distally; both the anterior and posterior cartilages are only present in males.

In stage-34 specimen ANSP 174692, the metapterygium is almost completely fused to the knob-like first metapterygial segment, with a fenestra (future foramen) remaining (Fig. 2e). Nearly all proximal radials are present and darkly-stained. Distal radials 1 and 2 have chondrified and distal radial 1 is wide compared to others. Distal radials are chondrifying following a general antero-posterior direction. The anterior clasper cartilage is taking a more elongated shape, and is closer to the basipterygial knob. The posterior clasper cartilage is extending distally.

**Figure 3:**
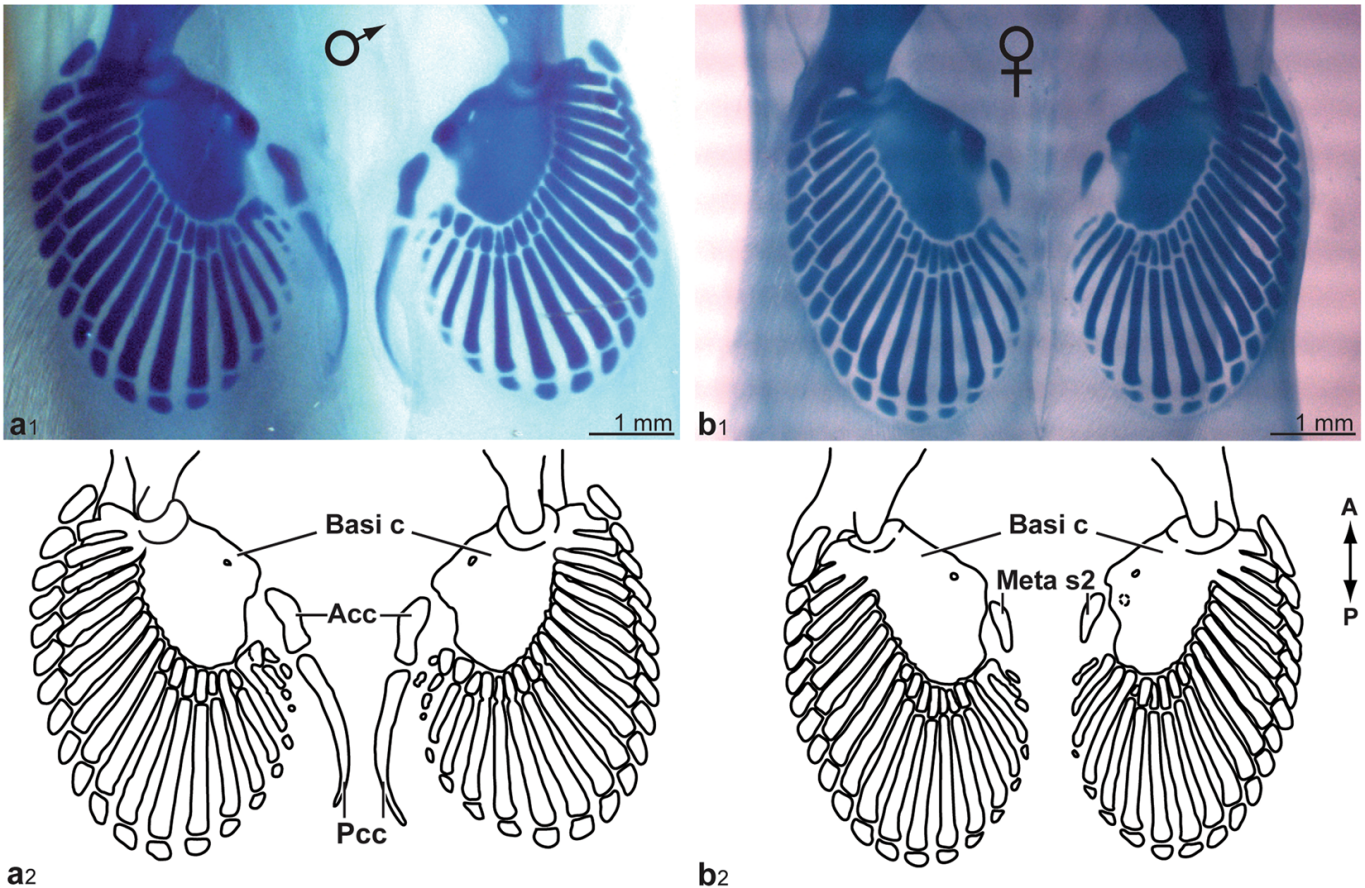
Pelvic fins of (**a**) male and (**b**) female of *Callorhinchus milii*, photographs and drawings of endoskeletal structures. (**a**) ANSP 174687, stage 35. (**b**) ANSP 174674, stage 35. Abbreviations: **Acc**, anterior clasper cartilage; **Basi c**, basipterygial complex; **Meta s2**, second metapterygial segment; **Pcc**, posterior clasper cartilage. All specimens are cleared and stained; cartilages are stained in blue.

In stage-35 specimens, the basipterygium is complete, formed by the fusion of the metapterygium and the first metapterygial segment (Fig. 3). The region of the basipterygium articulating with the pelvic girdle has taken the appearance of a rounded ridge. The first two middle radials (1–2) are longitudinally fusing together proximo-distally, and the proximal extremities of middle radials 1–4 fuse to the basipterygium to complete the basipterygial process. Distal radials are more lightly-stained in the posterior part of the fin (Fig. 3b).

In stage-35 male specimens (ANSP 174653, 174663, 174687), the anterior clasper cartilage is rounded proximally, tapers in the middle and widens distally, and articulates with the posterior clasper cartilage, which is thin, elongated, and slightly curved (Fig. 3a). In specimen ANSP 174687, the basipterygial process is complete and fused to the anterior region of the basipterygium articulating with the girdle (Fig. 3a). Proximal radials 18–19 have fused together and articulate with the concave posterior section of the basipterygium on the right fin, thus documenting fusion between anterior radials of the pelvic fin. All distal radials have chondrified, following an antero-posterior direction. A female specimen (ANSP 174674) shows only the second metapterygial segment, which is homologous to the anterior clasper cartilage; the proximal edge is rounded, whereas its distal edge is pointed (Fig. 3b).

Stage-36 pelvic fins (ANSP 174675) show a condition similar to that of the pectoral fins, where all elements appear more tightly associated (Fig. 2f). Two small rounded structures, similar to irregular elements, are present posterior to the last distal radial. The proximal edge of the anterior clasper cartilage is more rounded and forms a clear articulation with the concave posterior edge of the basipterygial process. The posterior clasper cartilage is thicker, and its anterior extremity forms a well-defined articulation with the posterior extremity of the anterior clasper cartilage (Fig. 2f).

### Morphological similarities

**Figure 4:**
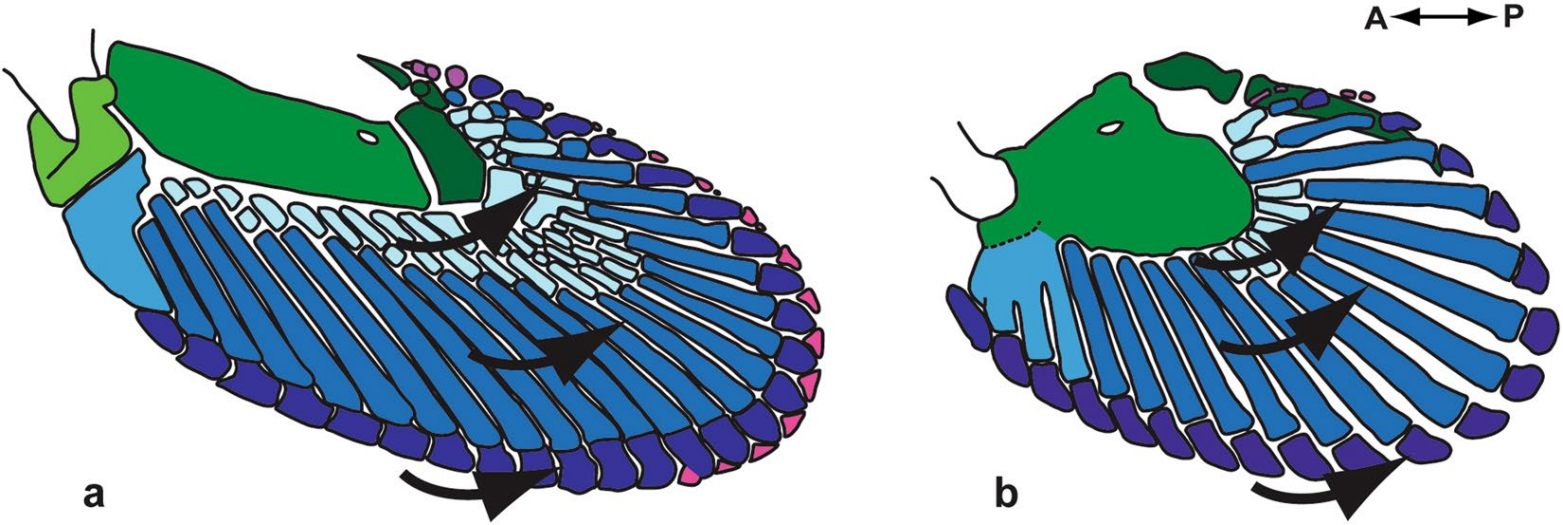
Concordance between the development of (**a**) pectoral and (**b**) pelvic fins elements in *Callorhinchus milii*. Similar elements are color-coordinated between paired fins. Arrows illustrate the direction of chondrification of basal and radial elements. Drawings based on stage-36 specimen ANSP 174675.

The morphology of the pectoral fins is highly similar to that of pelvic fins (Fig. 4). Both the pectoral and pelvic fins possess a metapterygial complex. In the pectoral and pelvic fins, the first metapterygial segment fuses with the metapterygium (and its lateral process in the pectoral fin) to form the metapterygial complex. Both pectoral and pelvic fins have metapterygial segments distal to the metapterygial complex. The pectoral fin includes an anterior radial element made of the fused anterior proximal and middle radials, whereas the pelvic fin includes a basipterygial process made of fused anterior middle radials. Both pectoral and pelvic fins have all three types of radials: proximal, median and distal. Both pectoral and pelvic fins have one row of middle radials and one row of distal radials (Fig. 4).

Minor differences between the later stages of pectoral and pelvic fins were also observed. Pectoral fins are more elongated and exhibit greater morphological complexity compared to the rounded pelvic fins. This complexity is characterized by pectoral fins having a greater number of elements (propterygium, radials) and differents classes of elements (e.g. interdistals) than pelvic fins. Interdistal and irregular elements, which appear towards the end of fin development (stage 35), are present in the pectoral fin but absent in the pelvic fin.

### Developmental similarities

The order in which the cartilaginous elements form in the pelvic fins is similar to that observed in the pectoral fins (Figs 1 and 2). The basal elements chondrify first, followed by the middle and proximal radials, whereas distal radials were the last to chondrify. The metapterygial segments and the clasper cartilages chondrify later in development, when the majority of radials are formed. The direction of formation of the mesenchymatous rods within the paired fins of stage-29 specimens was not observed. The direction of chondrification within the pelvic fins is similar to the one observed in pectoral fins (Figs 1 and 2). The metapterygial complex of the paired fins chondrifies following a proximo-distal direction of formation. Metapterygial segments, including the claspers, chondrify following a proximo-distal direction. Radials (proximal, middle and distal) present in the anterior part of the pectoral and pelvic fins are first to chondrify, followed by those in the middle and finally those in the posterior part, following an antero-posterior direction of formation. The formation of the anterior radial element and basipterygial process (from the fusion of individual radials) follows an antero-posterior and proximo-distal direction. The interdistal elements of the pectoral fins appear to be the only ones following a postero-anterior direction of formation. A general proximo-distal direction of chondrification is also observed in both the pectoral and pelvic fins; the first elements to chondrify are the basals, followed by the middle radials, proximal and finally the distal radials. The progression of chondrification in pectoral fins precedes that of the pelvic fins. Thus, an earlier stage of pectoral fin development (stage 31, Fig. 1c) is more similar to a later stage of pelvic fin development (stage 32, Fig. 2c).

## Discussion

We have documented the progression of chondrification for the pectoral and pelvic fins of a basal-lineage living gnathostome, the elephant shark *C*. *milii*. Previous interpretations suggested that chondrichthyan pectoral and pelvic fins were anatomically different from one another^5^, and that a high degree of similarity within their structural plan was either the exception or present as a secondary condition^27^. Pectoral and pelvic fins of basal gnathostomes were also proposed to be anatomically different from one another^28^ and to develop differently during early ontogeny^29^; similarity between them was hypothesized to be only found in sarcopterygians, tetrapods included^19, 28, 30, 31^. Our results contradict these previous interpretations and show that a high degree of similarity in the morphology and developmental patterning of the pectoral and pelvic appendages is also present in a chondrichthyan, the elephant shark *C*. *milii*.

The strong similarity in the morphology and concerted directions of endoskeletal patterning observed within *C*. *milii* paired fins suggest that the pectoral and pelvic fins are serial homologues, similar to paired limbs^2, 7, 8^. They are recognizable as serial homologues because they are variations of the same structural organization and share at least part of a developmental program^7, 8, 32^. This similarity suggests that the pectoral and pelvic fins correspond to a morphological and developmental module. A morphological module is a cohesive unit of organismal integration composed of hierarchically organized parts that can be recognized through anatomical similarities^33, 34^. A developmental module can be described in terms of recurrent anatomical direction of formation among serial elements^9, 15, 35, 36^. Thus, the high degree of morphological and developmental similarity suggests that the pectoral and pelvic fins in *C*. *milii* constitute morphological and developmental modules. The concerted molecular developmental patterns observed between the pectoral and pelvic fins of different species of chondrichthyans^11–13^ corroborates the presence of modules in paired fins. Other modules have been identified in the median and paired fins of fish^12, 35–37^, but none have been previously described which focus specifically on the anatomical structures in the paired fins of fishes. The modules described in *C*. *milii* are the first to be characterized using a detailed description of the patterning (progression of chondrification) of endoskeletal elements in the paired fins of a chondrichthyan, and in the paired fins of fishes in general.

Modules can be affected by several evolutionary processes such as dissociation, duplication and co-option^9^. Because modules are physical units that can be rearranged spatially, a duplicated module can be deployed to another position within an organism^7, 8, 38^. This is in agreement with the two classical hypotheses proposed for the emergence of paired fins: the lateral finfold^39–41^ and the gill arch^42^ hypotheses. Both suggest that pre-existing structures (lateral finfolds and gill arches, respectively) were transformed into pectoral fins first and then into pelvic fins. In accordance with the lateral finfold hypothesis, it has been suggested that the fin developmental mechanisms first evolved within a dorsal competence zone^43^, which is corroborated by the fact that median fins have appeared before paired fins in the fossil record^37, 44, 45^. This competence zone would then have been duplicated and co-opted to a novel area along the flank, eventually shifting to the lateral plate mesoderm (LPM) which produced fin buds and endoskeletal structures in the pectoral region first^43, 45^. This hypothesis is also corroborated by similar gene expression (*Hox*, *Tbx*) in the formation of median fins in the lamprey *Petromyzon marinus*, median fins in the shark *Scyliorhinus canicula* and tetrapod limbs^46^.

Horton *et al*.^47^ proposed co-option of an ancestral heart specifying *Tbx4*/*5* cluster for limb outgrowth, which resulted into distinct *Tbx5* and *Tbx4* genes associated with pectoral and pelvic appendages, respectively^48^. In agreement with this, *Tbx4*/*5* is limited to the heart region in the sea lamprey, whereas *Tbx5* is expressed in the lateral plate mesoderm of the pectoral region of gnathostomes^49^. *Tbx4* is expressed in the LPM of the pelvic region and is very similar to *Tbx5* ^3, 50^. Similar gene expressions are observed in the pectoral and pelvic appendages of fish (teleost, chondrichthyans) and tetrapods (birds, mammals) ^3, 7, 8, 11–13, 18, 50–53^. This similarity in the developmental mechanisms responsible for pectoral and pelvic appendage patterning supports their serial homology. See Supplementary Information for summary of gene expression in chondrichthyan paired fins.

As serial homologues, pectoral and pelvic appendages need not have identical patterning, especially given that nested cranial-caudal *Hox* gene expression has been implicated in regionalized specialization of the somatic lateral plate mesoderm^3^. Even though the genes responsible for appendage outgrowth and axis formation (among others) are fundamentally shared between pectoral and pelvic appendages ^3, 7, 8, 11–13, 18, 50–53^, changes in subsequent developmental regulation can produce morphological and developmental variations in homologous structures^2, 7, 8, 32^. Therefore, morphological and developmental similarities and differences observed within the paired fins of *C*. *milii* may be explained as follows. The generic Bauplan of the paired fins represents the module, which is subsequently modified at the pectoral level resulting in a greater number of endoskeletal elements, or at the pelvic level resulting in a reduced number of elements. Variation in the morphology and number of paired fin elements in zebrafish and chondrichthyans have been correlated with different *Shh* and retinoic acid expressions^11, 51, 52^. Claspers are another modification observed in the pelvic fin of male chondrichthyans, albeit sex-based, whose growth is promoted by a prolonged phase of *Shh* signaling in the pelvic fins^11, 51^.

Variations in the musculature and skeletal architecture of pectoral versus pelvic fins in gnathostomes has been used to argue that pectoral and pelvic appendages are not serial homologues^28, 30, 31^. The morphological and developmental similarity in tetrapod limbs was attributed to a convergence in the developmental programs for fish appendages prior to the appearance of tetrapods^30, 31^. Yet, work on phylogenetically successive taxa suggest a gradual change in the muscle formation processes of paired appendages, from the lack of muscle formation within the abaxial domain (lamprey^54^), to direct epithelial myotomal extensions (shark and chimaera^55^) to an intermediate mode where somitic cells extend from the somite towards their future position (paddlefish, zebrafish, lungfish^55^) to a fully derived mode in tetrapods^55^. These important evolutionary changes could lead to phenotypic variations in the identity and location of muscles of paired appendages, but their embryonic origin remains identical. The similar molecular controls in the anterior and posterior appendage outgrowth of elasmobranchs, teleosts, birds and mice support the notion that these appendages are evolutionary and developmentally homologous. This molecular similarity does not support the argument for convergence between the regulatory molecular patterns of pectoral and pelvic appendages just prior to the rise of the tetrapod condition. Needless to say, however, that more detailed descriptions of fin patterning are necessary from different chondrichthyan species to verify if the patterns observed for *C*. *milii* are similar for chondrichthyans in general. Also, more detailed developmental (gene expression, cell lineage tracing), morphological and paleontological data will be necessary to validate the duplication hypothesis.

Our results also support the hypothesis that the anterior radial element results from the fusion of anterior proximal and median radials^26^. On the other hand, the hypothesis that the propterygium and mesopterygium result from the fusion of radials^56^ was not validated in *C*. *milii* because the basal elements chondrified as single elements and not from the fusion of radials. The metapterygium of the pectoral fin appears to result from the fusion of two originally distinct basal elements. If this is the case, and that these basal elements were interpretated to represent a metapterygium and mesopterygium (as opposed to the metapterygium and a metapterygial segment as interpreted here), *C*. *milli* would thus initially show a tribasal pectoral fin condition, which has been suggested as the plesiomorphic condition for chondrichthyans^6, 19^.

## Conclusion

Knowledge of paired appendage morphological patterning, although quite extensive in derived sarcopterygians such as tetrapods, has remained limited in basal extant gnathostomes such as chondrichthyans. Our results based on endoskeletal fin patterning in a chondrichthyan support the notion that pectoral and pelvic fins are modules and serially homologous structures.

## Material and Methods

A total of 23 cleared and doubled-stained embryos^26^ of *Callorhinchus milii* (Holocephali: Callorhinchidae) from the Academy of Natural Science of Drexel University (ANSP), Philadelphia, Pennsylvania (USA) were used for this study. These embryos were originally caught and cleared and stained by Didier (1995)^26^. We used these specimens to assemble a growth series, based on the developmental stages that were assigned by Didier (1998)^57^, to describe the progress of chondrification. Specimens were observed and photographed using a digital camera mounted on a microscope (Olympus ZH10 research stereomicroscope and Olympus SZ50 dissecting scope). These photographs were then adjusted with filtering tools (brightness/contrast, exposure, gamma and invert colors) in Adobe Photoshop in order to display structures optimally. Drawings were made from the photographs. Description focuses on pectoral and pelvic endoskeletal elements in embryonic specimens of developmental stages 29–36^57^.

The sequence of skeletal element formation was ascertained by their ontogenetic appearance in relation to other skeletal elements^35, 36^. This sequence was also used to assess directionality in fin development, along with the degree of staining; elements that were more darkly stained were inferred to have appeared before lightly-stained ones in a given specimen. This is due to the fact that as development proceeds within a single specimen, tissue progressively forms and mature. Older cartilaginous structures will be in a more advanced state of development and have a greater number of glycosaminoglycans and proteoglycans stained by Alcian blue compared to younger ones^58^. Alcian blue staining is a standard laboratory method used to study cartilage formation in vertebrates, including chondrichthyans^59^.

Terminology follows Didier (1995)^26^; structures that were not identified previously are named herein. The developmental stages^57^, ANSP catalog numbers, total lengths and sex of specimens indicated for specimens are presented as Supplementary Information.

## Electronic supplementary material

Supplementary Information
