# Supplementary material for: Similarity of morphological composition and developmental patterning in paired fins of the elephant shark
Source: Sci Rep. 2017 Aug 30;7:9985. doi: 10.1038/s41598-017-10538-0 (PMC5577158; doi:10.1038/s41598-017-10538-0)
Supplement: Supplementary file 1 — Supplementary Information [file 41598_2017_10538_MOESM1_ESM.pdf]

Supplementary Information

Similarity of morphological composition and developmental patterning in paired fins of the elephant shark

Cyrena Riley[1], Richard Cloutier[1*] & Eileen D. Grogan[2]

Affiliation:

[1] Laboratoire de Biologie évolutive, Université du Québec à Rimouski, Rimouski, Québec, G5L 3A1, Canada

[2] Biology Department, Saint Joseph's University, Philadelphia, Pennsylvania, 19131, U.S.A.

Supplementary discussion

Horton *et al.*[47] proposed co-option of an ancestral heart specifying *Tbx4/5* cluster for limb outgrowth. In the process of cluster duplication, the distinct *Tbx4* and *Tbx5* genes associated with gnathostome pectoral (*Tbx5*) and pelvic (*Tbx4*) development would have arisen in the posterior (non cardiac) LPM[47]. Tulenko *et al.*[54] confirmed that the dHand transcription factor of early LPM is expressed in the branchial, cardiac and posterior LPM of the lamprey *Lethenteron*.

Adachi *et al.*[49] evaluated *Tbx4/5* expression in the lamprey *P. marinus* and *Tbx5* expression in the skate *Leucoraja erinacea* and zebrafish (with paired fins) and mouse (with paired limbs). As predicted by Horton *et al.*, this work revealed *Tbx4/5* expression in sea lamprey embryos was limited to the heart region. Expression of the *Tbx5* domain into the lateral plate mesoderm of the pectoral region was only present in paired finned and limbed species and its expression was controlled by the fin enhancer, CNS12[49]. A recent summary of other relevent research now confirms the lamprey has a *Tbx5* ortholog expression domain in the anterior LPM but not in the posterior LPM and that the latter is not separated into distinct somatic and visceral aspects[3]. In comparison, however, gnathostomes, have a relatively extended *Tbx5* expression domain and this occurs in the posterior LPM, specifically in the anterior appendage forming field of the somatopleure[3,49,54].

Horton *et al.*'s proposition of co-option and duplication is supported by other molecular studies, revealing *Tbx4* is very similar to *Tbx5*. *Tbx4* expression in the more caudal aspect of the lateral plate mesoderm stimulates pelvic appendage outgrowth in the shark, teleost, chick and mouse [3,18,48] and is controlled in part by the action of *Pitx1* which is associated with hindlimb identity[3,32]. Further, substitution of *Tbx5* with *Tbx4* in transgenic mice results in forelimb initiation[3,50].

Tbx4 and *Tbx5* are also present in the paired fins of sharks[18], and *Pitx1* has been

confirmed in catsharks[60]. Similar gene expression are also observed between the pectoral and pelvic fins of chondrichthyans[11-13,18,51-53] (Fig. S2, revised from Fig. 4 in Tanaka 2016). This similarity in the developmental mechanisms responsible for paired appendage patterning supports their serial homology.

References

3 Tanaka, M. Developmental mechanism of limb field specification along the anterior–posterior axis during vertebrate evolution *J. Dev. Biol.* **4**, doi:10.3390/jdb4020018 (2016).

11 Dahn, R. D., Davis, M. C., Pappano, W. N. & Shubin, N. H. *Sonic hedgehog* function in chondrichthyan fins and the evolution of appendage patterning. *Nature* **445**, 311-314 (2007).

12 Freitas, R., Zhang, G. J. & Cohn, M. J. Biphasic *Hoxd* gene expression in shark paired fins reveals an ancient origin of the distal limb domain. *PLoS ONE* **2**, doi:10.1371/journal.pone.0000754 (2007).

13 Yonei-Tamura, S. *et al.* Competent stripes for diverse positions of limbs/fins in gnathostome embryos. *Evol. Dev.* **10**, 737-745 (2008).

18 Tanaka, M. *et al.* Fin development in a cartilaginous fish and the origin of vertebrate limbs. *Nature* **416**, 527-531 (2002).

32 Ouimette, J.-F., Lavertu Jolin, M., L'Honoré, A., Gifuni, A. & Drouin, J. Divergent transcriptional activities determine limb identity. *Nat. Commun.* **1**, 35 (2010).

47 Horton, A. C. *et al*. Conservation of linkage and evolution of developmental function within the *Tbx2/3/4/5* subfamily of *T-box* genes: implications for the origin of vertebrate limbs. *Dev. Genes Evol.* **218**, 613-628 (2008).

48 Gibson-Brown, J. J. *et al*. Evidence of a role for *T-box* genes in the evolution of limb morphogenesis and the specification of forelimb/hindlimb identity. *Mech. Dev.* **56**, 93-101 (1996).

49 Adachi, N., Robinson, M., Goolsbee, A. & Shubin, N. H. Regulatory evolution of *Tbx5* and the origin of paired appendages. *Proc. Natl. Acad. Sci. USA.* **113**, 10 115-10 120 (2016).

50 Minguillon, C., Del Buono, J. & Logan, M.P. *Tbx5* and *Tbx4* are not sufficient to determine limb-specific morphologies but have common roles in initiating limb outgrowth. *Dev. Cell.* **8,** 75-84 (2005).

51 O'Shaughnessy, K. L., Dahn, R. D. & Cohn, M. J. Molecular development of chondrichthyan claspers and the evolution of copulatory organs. *Nat. Commun.* **6**, doi: 10.1038/ncomms7698 (2015).

52 Sakamoto, K. *et al.* Heterochronic shift in *Hox*-mediated activation of *Sonic hedgehog* leads to morphological changes during fin development. *PLoS ONE* **4,** doi.org/10.1371/journal.pone.0005121 (2009).

53 Onimaru, K. Marcon, L., Musy, M., Tanaka, M. & Sharpe, J. The fin-to-limb transition as the re-organization of a Turing pattern. *Nat. Commun.* **7**, doi:10.1038/ncomms11582 (2016).

54 Tulenko, F. J. *et al*. Body wall development in lamprey and a new perspective on the origin of vertebrate paired fins. *Proc. Nat. Acad. Sci. USA* **110**, 11 899-11 904 (2013).

60 Rasch, L. J. *et al*. An ancient dental gene set governs development and continuous regeneration of teeth in sharks. *Dev. Biol.* **415**, 347-370 (2016).

Supplementary table S1. Developmental stage, ANSP catalog number, total length (mm) and sex of *Callorhinchus milii* specimens.

Stage	ANSP catalog number	Total length (mm)	Sex
29	174694	56.25	Undetermined
29	174695	62.10	Undetermined
29	174690	66.75	Undetermined
30	174667	69	Undetermined
30	174665	69.3	Undetermined
30	174658	69.5	Undetermined
30	174656	71.95	Undetermined
31	174693	72.75	Undetermined
31	174689	79.3	Undetermined
31	174661	79.4	Undetermined
32	174696	74.05	Undetermined
32	174688	75.90	Undetermined
33	174659	78.7	Undetermined
33	174682	80.97	Undetermined
33	174691	83.6	Undetermined
34	174711	87	Undetermined
34	174692	89	Undetermined
34	174712	98	Undetermined
35	174663	95	Male
35	174674	100	Female
35	174687	101.6	Male
35	174653	103.85	Male
36	174675	110	Male

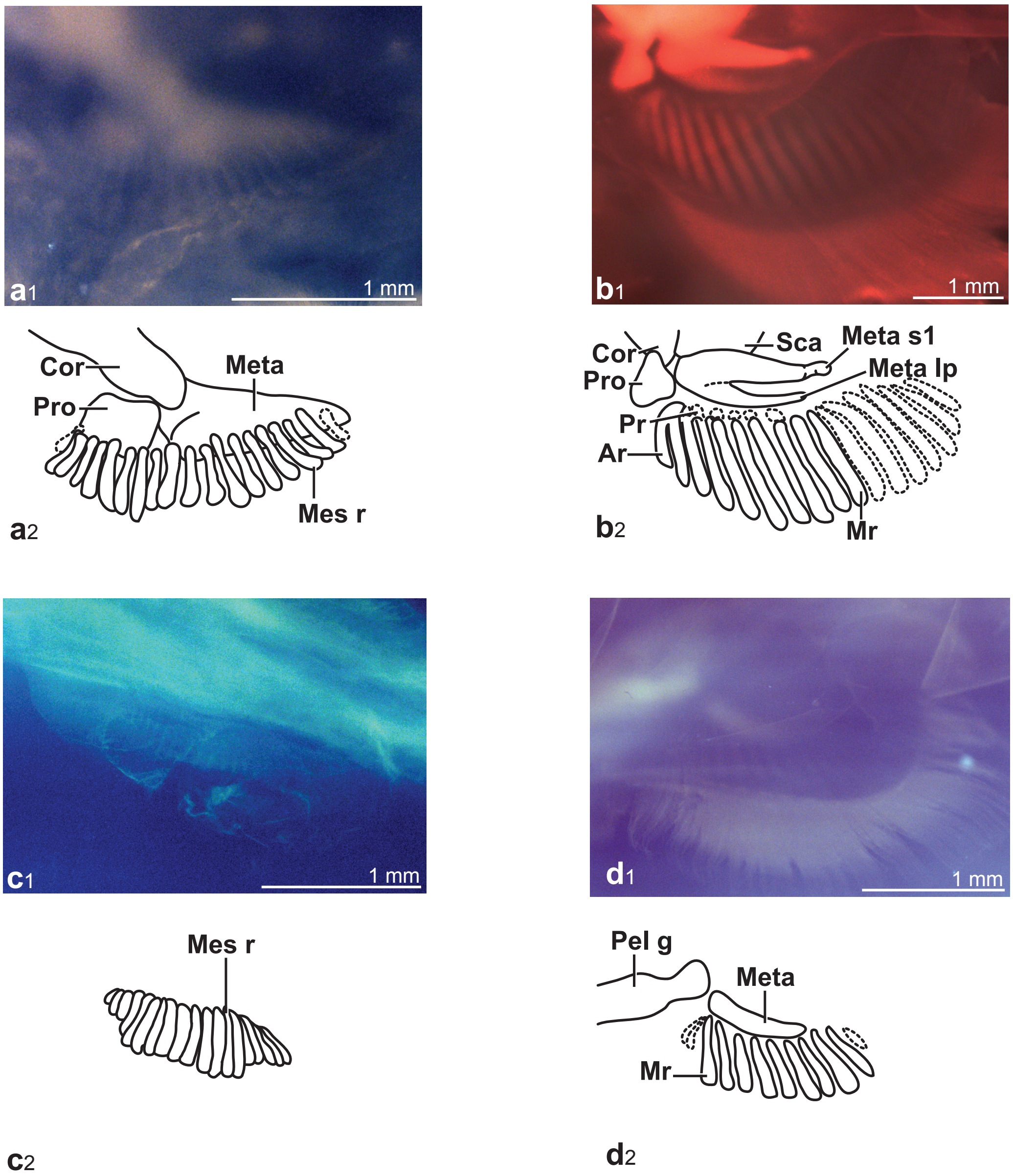

Supplementary Figure S1. ***Callorhinchus milii*** **pectoral (a, b) and pelvic (c, d) fins, photographs and drawings of endoskeletal structures.** Pectoral fins: (**a**) ANSP 174667, stage 30; (**b**) ANSP 174661, stage 31. Pelvic fins: (**c**) ANSP 174690, stage 29; (**d**) ANSP 174661,stage 31. Abbreviations: **Ar**, anterior radial element; **Cor**, coracoid; **Dr**, distal radial; **Mes r**, mesenchymatous rod; **Meta**, metapterygium; **Meta lp**, metapterygial lateral process; **Meta s1**, first metapterygial segment; **Mr**, middle radial; **Pel g**, pelvic guirdle; **Pr**, proximal radial; **Pro**, propterygium; **Sca**, scapula. All specimens are cleared and stained. Colors in the photographs were inverted and optimized using the Adobe Photoshop invert and exposure tools in order to enhance endoskeletal structures.

Paired fins module

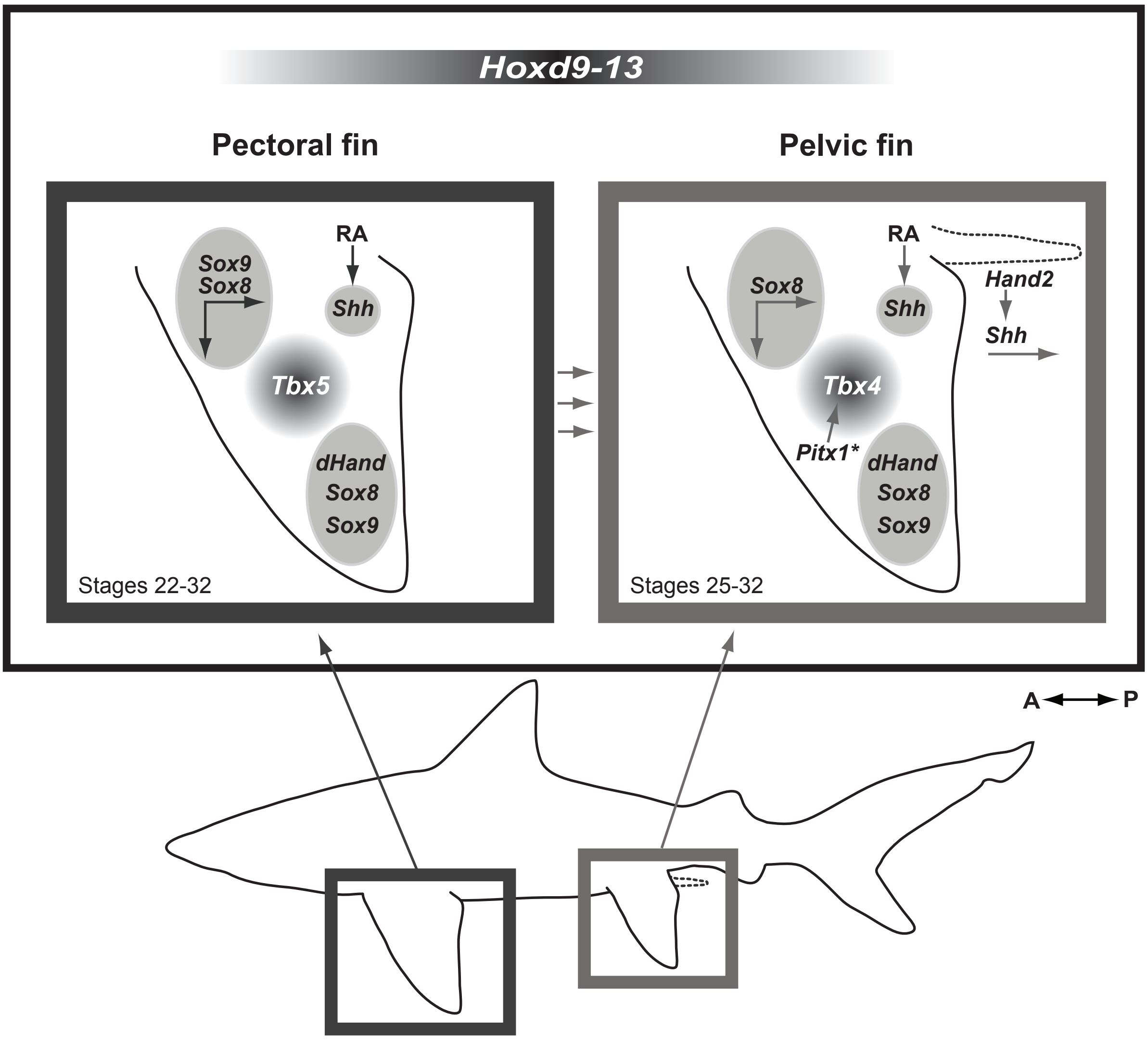

Supplementary Figure S2 (revised from Fig. 4 in Tanaka 2016). Genes implicated in the development of chondrichthyan paired fins. The serial homology of pectoral and pelvic fins is supported by the same genes being expressed similarly in pectoral and pelvic fins. In the pelvic fins of males a sustained expression of *Shh* maintained by *Hand2* is associated with the formation of claspers. *Tbx5* is associated with pectoral fin initiation whereas *Tbx4* is associated with pelvic fin initiation, possibly modulated by *Pitx-1*. **Pitx-1* activity has been confirmed in catshark, associated with teeth formation.[60]
